# Highly Conductive and Reusable Cellulose Hydrogels for Supercapacitor Applications

**DOI:** 10.3390/mi14071461

**Published:** 2023-07-21

**Authors:** Nujud Mohammed Badawi, Khalid Mujasam Batoo, Ramesh Subramaniam, Ramesh Kasi, Sajjad Hussain, Ahamad Imran, Muthumareeswaran Muthuramamoorthy

**Affiliations:** 1 Centre for Ionics, Department of Physics, Faculty of Science, Universiti Malaya, Kuala Lumpur 50603, Malaysiaramesh@um.edu.my (R.S.); 2 Department of Physics, Faculty of Science, College of Science, University of Hafr Al-Batin, Hafer Al-Batin 39921, Saudi Arabia; 3 King Abdullah Institute for Nanotechnology, King Saud University, P.O. Box 2455, Riyadh 11451, Saudi Arabia; aimran@ksu.edu.sa (A.I.);; 4 Graphene Research Institute and Institute of Nano and Advanced Materials Engineering, Sejong University, Seoul 143-747, Republic of Korea; shussainawan@gmail.com

**Keywords:** Na-Alginate/PEDOT:PSS, self-healing, hydrogel, ionic conductivity, flexible supercapacitor, electrolyte

## Abstract

We report Na-Alginate-based hydrogels with high ionic conductivity and water content fabrication using poly (3,4-ethylene dioxythiophene) (PEDOT): poly (4-styrene sulfonic acid) (PSS) and a hydrogel matrix based on dimethyl sulfoxide (DMSO). DMSO was incorporated within the PEDOT:PSS hydrogel. A hydrogel with higher conductivity was created through the in-situ synthesis of intra-Na-Alginate, which was then improved upon by H_2_SO_4_ treatment. Field emission scanning electron microscopy (FESEM) was used to examine the surface morphology of the pure and synthetic hydrogel. Structural analysis was performed using Fourier-transform infrared spectroscopy (FTIR). Thermogravimetric analysis (TGA), which examines thermal properties, was also used. A specific capacitance of 312 F/g at 80 mV/s (energy density of 40.58 W/kg at a power density of 402.20 W/kg) at 100 DC mA/g was achieved by the symmetric Na-Alginate/PEDOT:PSS based flexible supercapacitor. The electrolyte achieved a higher ionic conductivity of 9.82 × 10^−2^ and 7.6 × 10^−2^ Scm^−1^ of Na-Alginate and a composite of Na-Alginate/PEDOT:PSS at 25 °C. Furthermore, the supercapacitor Na-Alginate/PEDOT:PSS//AC had excellent electrochemical stability by showing a capacity retention of 92.5% after 3000 continuous charge–discharge cycles at 10 mA current density. The Na- Alginate/PEDOT:PSS hydrogel displayed excellent flexibility and self-healing after re-contacting the two cut hydrogel samples of electrolyte for 90 min because of the dynamic cross-linking network efficiently dissipated energy. The illumination of a light-emitting diode (LED) verified the hydrogel’s capacity for self-healing.

## 1. Introduction

**Figure 1:**
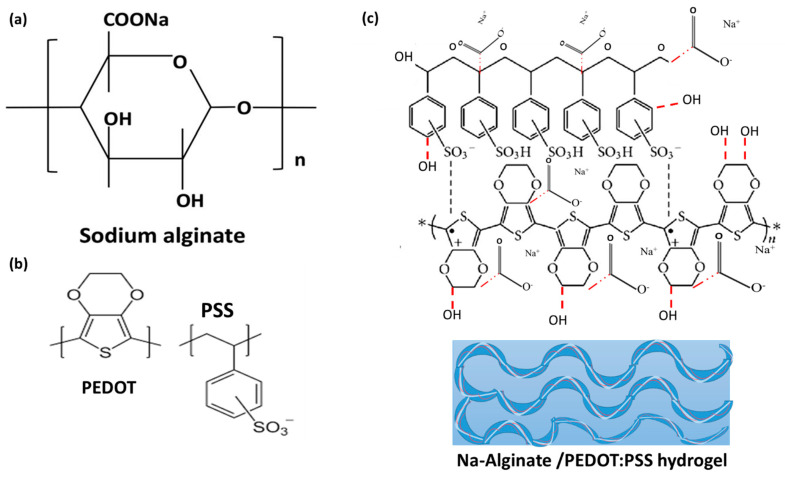
Structure units of (**a**) Alginate molecule, (**b**) PEDOT:PSS molecule, and (**c**) Na-Alginate/PEDOT:PSS molecule.

Hydrogels contain a significant amount of water in their structures and are widely used in the fields of tissue engineering, biosensors, and actuators’ range of biomedical, pharmaceutical, battery, and supercapacitor applications [1]. Supercapacitors, a type of flexible energy storage device, have drawn a lot of attention because they enable wearable electronics. A new kind of supercapacitor that can function in conditions of excessive bending, torsion, and expansion is known as a solid-state supercapacitor. A significant challenge exists in creating a flexible supercapacitor with high electrochemical performance and super flexibility in a solid state. The characteristics of the electrolyte materials, electrode materials, and device configuration all affect the performance of flexible supercapacitors (SCs) [2]. Fast charge and discharge rates, high energy density, ease of manufacture, and low cost are some of the characteristics of flexible solid-state super capacitors (ASSSC), which have promising uses as power sources for multipurpose portable and wearable electronic devices. The configuration and flexibility of the devices are governed by the mechanical strength of the active electrodes and electrolyte. Thus, it is anticipated that supercapacitors will exhibit exceptional mechanical flexibility and be able to absorb high levels of stress in demanding real-world applications [1]. Alginic acid is a naturally occurring polyanionic, non-toxic, biocompatible, non-immunogenic, and biodegradable linear carbohydrate biopolymer derived from seaweed. Sodium alginate (SA) is the sodium salt of alginic acid. D-mannuronic acid (M-block) and L-guluronic acid (G-block) are the two monomeric components of SA. SA is frequently used in biomedical gels because of its biocompatibility, biodegradability, good gel-forming properties, and low cost [2]. Some synthetic polymers, such as poly(ethylene glycol) (PEG), PEDOT:PSS p(3,4-ethylene dioxythiophene) (PEDOT): poly(4-styrene sulfonic acid) (PSS), poly(hydroxyethyl methacrylate) (PHEMA) [3], and poly(N-isopropyl acrylamide) (PNIPAM) [4], are used in the production of 3D hydrogels. Conductive hydrogels have received much attention because they possess two main components: a tissue-mimicking hydrogel component and an electrically conductive component that provides the electrical stimulus. However, removing the unpolymerized monomer residues, which are extremely toxic in biomedical and pharmaceutical applications, is very challenging [4]. Hydrogels obtained by combining natural polymers, such as chitosan, starch cellulose, collagen, gelatin, and alginate [5], are of great interest in many applications and semi-synthetic polymers for the fabrication of hydrogels [6,7]. These materials are biodegradable and biocompatible. Water-soluble sodium alginate is used to prepare the hydrogel by the wet-spinning process. Alginate is a linear polymer consisting of a natural anionic polysaccharide derived from brown algae, having 1,4-beta-D-manuronic acid, and alpha-L-guluronic acid, or β-(1 → 4) D-mannuronic acid (M) and α-(1 → 4))-linked L-guluronic acid (G) residues (Figure 1) [8,9], where one part of it can be divided into three parts: “M blocks, which are *rich in mannuronic acid residues*”; “G blocks, which are *rich in guluronic acid residues*”; and “MG block, that is *rich in both*” [10,11] (Figure 1). Nonwovens from alginate hydrogels are used in many applications due to their biocompatibility, biodegradability, and non-toxic properties [12]. Na-Alginate is also used to solve the problem of using nano-filling materials. By physically blending conductive polymers like PEDOT:PSS into the hydrogel, which causes the hydrogel to crosslink in the presence of the conductive polymer and weaken the conductive hydrogel’s mechanical properties, hydrogel blending systems are frequently less bonded [13]. The electrical performance of the conductive hydrogel is decreased by the discrete domain structure of the conductive polymers distributed within the hydrogel matrix. Due to issues with blending systems, the bulk structure’s desired physical characteristics are provided by the conductive hydrogel, which in turn causes the hydrogel’s electrical conductivity to be reduced by the polymerized conductive polymer. The hydrogel (Na-Alginate/PEDOT:PSS) could combine the benefits of Na-Alginate and PEDOT:PSS [14].

The hydrogel can repair the damage it has sustained thanks to its ability to self-healing, maintaining its fundamental properties and functions and, ultimately, extending the useful lives of the materials. The self-healing properties of polymeric materials can be divided into extrinsic and intrinsic self-healing depending on whether the self-healing component is inserted into the polymer or the original component is in the polymer matrix. An electron-poor hydrogen atom interacts with an electron-rich species over a short distance to form a hydrogen bond, a kind of physical interaction [15]. Heating can destroy the hydrogen bond but at a specific temperature, it can also regenerate. The material can produce self-healing effects thanks to this reversible effect [15]. The mechanical properties of hydrogels may be weakened by hydrogen bonding because of their inherent weakness, making them susceptible to competition with the nearby water molecules.

In the present work, the electrically conductive hydrogels fabricated by in situ polymerization of PEDOT:PSS within the pre-cross-linked Na-Alginate hydrogel were studied. Due to its excellent biocompatibility, high flexibility, and affordable price, the Na-Alginate hydrogel was chosen as the primary matrix, and PEDOT:PSS was chosen due to its biocompatibility and electrical conductivity. Strong hydrogen bonding relationships cause longer amylose molecules (and starch, which has more amylose) to gel into a stiff substance. Because they are more similar to amylose, longer-branched amylopectin molecules are more likely to produce strong hydrogels. While structural analysis was carried out using Fourier Transform Infrared Spectroscopy (FTIR), surface morphology was examined using Field Emission Scanning Electron Microscopy (FESEM). The supercapacitor was fabricated using a composite hydrogel electrolyte and the electrochemical properties were investigated using cyclic voltammetry (CV) techniques, and the GCD measurement was carried out at several potential windows between 0 and 0.8 V. Galvanostatic charge-discharge (GCD) at a current density of 10 mA for 3000 continuous charge-discharge cycles was used to test the supercapacitor’s cycling stability. A conductive Na-Alginate/PEDOT:PSS hydrogel with excellent adhesive and self-healing properties was created by numerous interactions between imine bond hydrogen bonds. Illuminating of a light-emitting diode (LED) allowed researchers to test the composite hydrogel electrolyte’s and the supercapacitor’s capacity for self-healing.

## 2. Materials and Methods

### 2.1. Materials

Powdered sodium alginate (Mn = 357,475, Mn/Mw = 1.392, M/G = 0.32) was purchased from Sigma Aldrich from the United States. Poly (3,4-ethylene dioxythiophene):poly (styrene sulfonate) (PEDOT:PSS) was bought from Osila, USA. Starch from wheat (C_6_H_10_O_5_)_n_ containing moisture content of about 8.8–11.5%, protein ≤ 0.3%, and particle size ≥95% (−120 mesh) was obtained. High amylopectin content in starch makes it easier to graft more acrylonitrile side chains, whereas high amylose content in starch decreases the grafting ratio of acrylonitrile. Dimethyl sulfoxide DMSO (CH_3_)_2_SO (assay 99.9%; Sigma Aldrich (Gillingham, UK)) and sulfuric acid H_2_SO_4_ were purchased from Sigma Aldrich, UK. The activated carbon (surface area: 1800–2000 m^2^/g, particle size: 5–20 m) coating was applied to the graphite conductive substrate, which was used as an electrode. This substrate was purchased from Sigma Aldrich. The solvent used was deionized water (DI).

### 2.2. Fabrication of Electrode

A total of 0.4 g of activated carbon was prepared in 30 mL deionized (DI) water and mixed under constant stirring for 1 h at 100 °C. Then, 0.5 mL of DMSO was added dropwise into the precursor solution under constant stirring. The activated carbon electrode was prepared by coating the slurry on a graphite sheet using the brushing method and then dried in an air oven at 100 °C for 30 min. The electrode was used to fabricate a supercapacitor.

### 2.3. Preparation of Na-Alginate/PEDOT:PSS Blended Hydrogel

**Figure 2:**
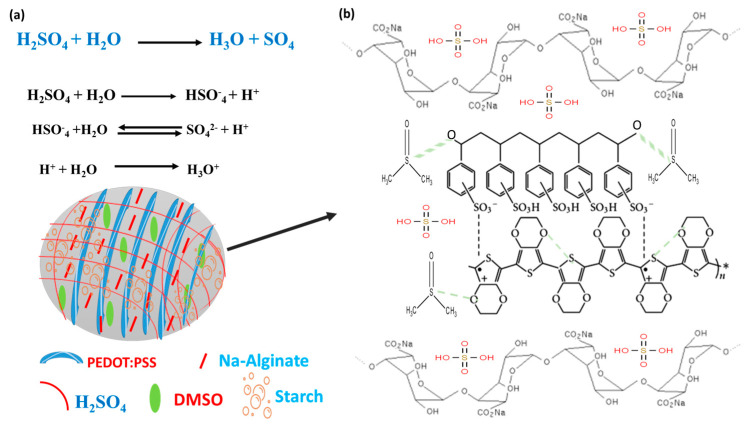
(**a**) Sulfuric acid (H_2_SO_4_) dissociates in water and (**b**) Na-alginate/PEDOT:PSS composite hydrogel electrolyte synthesis mechanism.

To prepare the hydrogel, 0.4 g of Na-Alginate/50 mL water was added to the beaker and 1 g starch was added to the solution. Further, 1 mL of DMSO and 3 mL PEDOT:PSS were added to the solution. The solution was then poured into glass tubes. The polymerization reaction was performed in tubes for 2 h at 60 °C, which was initiated by adding 4 mL solution of H_2_SO_4_. The reaction is best thought of as forming hydronium ions, by H_2_SO_4_ + H_2_O → H_3_O^+^ + HSO, where the strong linkages called covalent bonds hold together the hydrogen and oxygen atoms of individual H_2_O molecules as shown in Figure 2a. The hydrogels were fabricated with different DMSO and H_2_SO_4_ ratios as summarized in Table 1 and Figure 2b. The chemical structure of PEDOT:PSS is a polymer electrolyte consisting of positively charged conjugated PEDOT and negatively charged saturated PSS. PSS is a polymer surfactant, which helps disperse and stabilize PEDOT in water and other solvents. PEDOT:PSS is the most successful conducting polymer in terms of practical applications. The symbol * represents the point where the polymer linkage or bridging takes place to form chains.

### 2.4. Characterization

Field emission scanning electron microscopy (FESEM, Quanta FEG 450(E2 Building, Innovation Industrial Park II, High-tech District, Hefei, Anhui, China)) was used to examine the surface morphology of the synthesized material while operating at an accelerating voltage of 5 kV. Prior to FESEM analysis, the samples were coated in gold to create a conductive surface. Using a Nicolet iS50 FTIR spectrometer from Thermo Fisher Scientific Co. (Waltham, MA, USA), structural characterization of the pure and synthesized hydrogel was carried out in the frequency range of 500 to 4000 cm^−1^. Mechanical tests and the uniaxial tensile tests were performed with a tensile tester (Shimadzu AGS-X series equipped) at room temperature with a crosshead speed of 100 mm min^−1^. The dimensions of each hydrogel were cut into a dumbbell shape, which is 1 cm by 1 cm by 0.56 mm. From the slope of the stress-strain curves (e = 5% to 22%), the Young’s modulus was calculated. The area of the stress–strain curve was integrated to determine the fracture energy (toughness). The Na-Alginate and Na-Alginate/PEDOT:PSS hydrogels were wrapped in plastic for the subsequent loading–unloading tests to prevent water volatilization. The region between the loading–unloading curves served as the basis for calculating the dissipated energy (hysteresis).

### 2.5. Electrochemical Impedance Spectroscopy (EIS)

A Hioki 3532-50 LCR HiTESTER impedance spectrophotometer (Tech-Rentals (M) Sdn Bhd 29, Jalan Serendah 26/39, iParc 2, Seksyen 26,40400 Shah Alam Selangor) was used to estimate the ionic conductivity (σ) of the Na-Alginate/PEDOT:PSS hydrogel electrolyte in the frequency range of 40 Hz to 80 MHz [12,16]. The bulk resistance was calculated using the slope of the complex impedance plot [13]. The following equation was applied to impedance spectra to calculate the ionic conductivity [14].

(1) $$\sigma =\frac{L}{R_{b}\cdot A}$$

where *L* is the electrode distance (cm), *R_b_* is the bulk resistance (Ω), and *A* is the effective area of the electrode (cm^2^).

### 2.6. Electrochemical Studies

To prepare the electrochemical cells, which used Na-Alginate/PEDOT:PSS hydrogel as the electrolyte, the composite polymer hydrogel was sandwiched between graphite electrodes coated with activated carbon [15]. The characterization of the materials coated with graphite electrodes includes the measurement of physical properties and electrochemical properties. The electrochemical properties can be tested using the cyclic voltammetry (CV) method and galvanostatic charge–discharge (GCD) analysis. The CV method was used to find out the value of the specific capacitance of the supercapacitor, while GCD testing was performed to determine the charging and discharging properties of the fabricated capacitor of the cotton double-layer electrolytic cells at a specific current density and to determine the electrochemical behavior of the cotton double-layer electrolytic cells [16]. GCD measurement of the cotton double-layer electrolytic cells was carried out at the current densities of 3 to 100 mV/s with a potential window of 0.8 V. The specific capacitance (*C_sp_*) value of the supercapacitor was calculated through the following formula:

(2) $$C_{sp}=\frac{I_{c}-I_{d}}{S\cdot m}$$

where the symbols *C_sp_*, *I*, *s*, and *m* denote the specific capacitance, current density (Am^−2^), scan rate (mVs^−1^), and mass of electrode (g), respectively. The relationship between capacitance and the surface area is theoretically expressed using the following relation [17]:

(3) $$\frac{\varepsilon }{\pi \delta^{4}}=\frac{C}{A\;}$$

where *C* is the capacitance, *A* is the electrode’s surface area, and ε is the electrolyte’s dielectric constant, and *δ* is the distance from the surface of the electrode to the center of the ionic layer. The energy density *E* (Wh/kg) and power density *P* (W/kg) were also determined from the data obtained from GCD studies using Equations (4) and (5) [18]:

(4) $$E=\frac{C\cdot V^{\frac{2}{3.6}}}{2}$$

and

(5) $$P=\frac{E\cdot 3600}{t}$$

where ‘*E*’ is specific energy (Wh/kg), ‘*C*’ is the specific capacitance, ‘*V*’ is the potential window (V), ‘*P*’ is power density (W kg^−1^), and ‘*t*’ is the discharge time (s). Cyclic stability was tested to determine the stability of the supercapacitor upon multiple charging–discharging cycles. The cyclic stability of the device was tested using GCD at a current density of 10 A/g for 3000 continuous charge–discharge cycles.

### 2.7. Device Preparation

**Figure 3:**
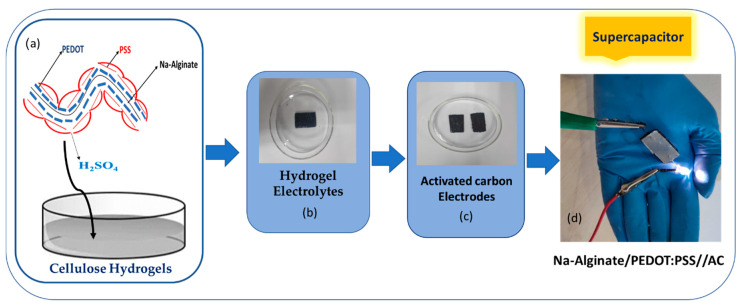
(**a**) Na-Alginate/PEDOT:PSS hydrogel as an electrolyte; (**b**) the graphite conductive substrate as an electrode; (**c**) fabricated supercapacitor; and (**d**) powering up a light-emitting diode (LED).

A supercapacitor was prepared by assembling the solid-state hydrogel electrolyte and electrodes as the positive and negative electrodes, respectively. The device was manufactured using an activated carbon electrode conductive substrate as an electrode and a Na-Alginate/PEDOT:PSS hydrogel as an electrolyte. The hydrogel was cut into a square shape and used as the electrolyte, and it was sandwiched between two electrodes based on activated carbon. The supercapacitor cell was connected to a copper wire, and a voltage of 9 V was applied to verify the electrochemical performance and reliability of the device for electronic applications [19]. The schematic of the supercapacitor is shown in Figure 3.

## 3. Results and Discussion

### 3.1. Synthesis of Composite Hydrogel Electrolytes

Na-Alginate (PEDOT:PSS) was prepared by redox polymerization and covalent linkage of the corresponding groups. As shown in Figure 2b, an environment-friendly cross-linked gene network was formed through the reaction of the carboxyl group (–COOH) of the alginate and C–C bonds on the thiophene rings in terms of PEDOT:PSS content. By forming these bonds, degradation defects and low mechanical strength of alginates can be controlled based on ionic bonds [20]. PEDOT:PSS can give the hydrogel excellent electrical properties due to its conductive nature [21]. After mixing all the reactants, the Na-alginate/PEDOT:PSS solution gradually became a hydrogel and turned bluish-black, which suggests that the redox polymer formed [22].

### 3.2. Morphologies of the Na-Alginate/PEDOT:PSS Hydrogels

**Figure 4:**
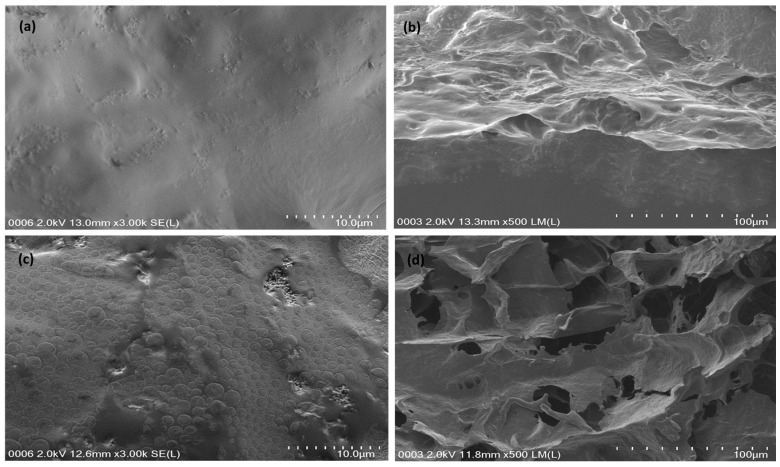
FESEM images of (**a**,**b**) Na-Alginate and (**c**,**d**) Na-Alginate/PEDOT:PSS Hydrogels.

The microstructures of Na-Alginate and Na-Alginate/PEDOT:PSS hydrogels are shown in Figure 4. In particular, the pores in the Na-Alginate hydrogel are large, irregular with thick walls, and heterogeneous, as shown in Figure 4a,b. The Na-Alginate hydrogels have large, erratic, and inhomogeneous slices for pore shapes [23]. The chemically cross-linked Na-Alginate hydrogels produced smooth surfaces with small pore sizes, as shown in Figure 4b, in comparison to chemically cross-linked hydrogels obtained by blending chitosan, as shown in Figure 4d. Meanwhile, pores in the Na-Alginate/PEDOT:PSS hydrogel are small, regular, and with homogeneous thin walls, as shown in Figure 4c,d. When the alginate/gelatin solution is treated with PEDOT, the following happens:PSS [20]. The morphologies of chemically cross-linked Na-Alginate and PEDOT:PSS hydrogels blended with chitosan and alginate indicated the formation of a smooth and porous surface due to cross-linking and crystallization of water during the formation of PEDOT:PSS with blended Na-Alginate hydrogels via the cross-linking process (Figure 4c) [18]. However, it has been discovered that the type of PEDOT:PSS in Na-Alginate polymer hydrogels and the type of cross-linking have no bearing on the size of pores and their distribution on the surface of hydrogels. Additional treatment with H_2_SO_4_ makes the cross-linkage even stronger. As a result, PEDOT:PSS-treated gelatin hydrogels have an impressive relative density [24].

### 3.3. FTIR Analysis

**Figure 5:**
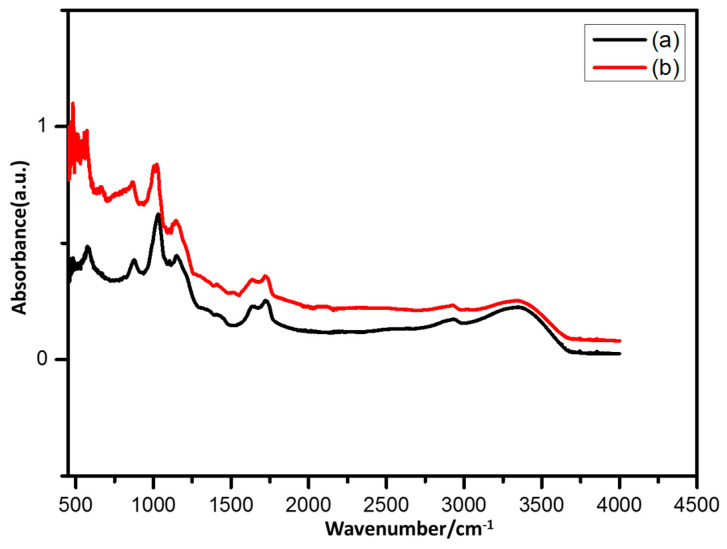
FTIR spectra of (a) Na-Alginate hydrogel, and (b) Na-Alginate/PEDOT:PSS hydrogel, respectively.

Na-Alginate hydrogel and Na-Alginate/PEDOT FTIR spectra, Figure 5 presents the PSS hydrogel. The peak at 3450 cm^−1^ in the alginate hydrogel’s FTIR spectrum is attributed to the –OH stretching vibrations. For the synthesized Na-Alginate/PEDOT:PSS hydrogel, the peaks at 3450 cm^−1^ are due to the partially overlapping stretching vibrations of the -OH groups in the PEDOT:PSS and -NH groups, which are in the gel state. These peaks represent the O–H bonds of the confined water molecules. The peaks at 1623 cm^−1^ and 1420 cm^−1^ are attributed to the vibration of symmetric and asymmetric stretching of the bonds of COO– groups due to the presence of Na-Alginate, and the peaks at 2606 cm^−1^ and 2400 cm^−1^ are assigned to the stretching vibrations caused by saturated –CH bonds [24]. The peak at 1060 cm^−1^ is due to the stretching vibrations of C–O–C groups, and the peak at 1010 cm^−1^ is due to the O–C=O stretching vibrations of Na-Alginate.

When comparing the spectrum of the FTIR alginate hydrogel with the composite PEDOT:PSS, some peaks are noted to disappear [25]. In addition, all electrodes also confirmed the presence of Na-Alginate hydrogel bonds, as shown in Figure 5. The expansion of vibrations of O–C=N groups, which is produced by the chemical reaction of –CHO in oxidised starch and H_2_N-OH in wheat starch, is responsible for the peak at 1620 cm^−1^ in the Na-Alginate/PEDOT:PSS spectrum. Additionally, the peak at 1326 cm^−1^, which is from Na-Alginate/PEDOT:PSS, is attributed to Na-Alginate with bending oscillations for the –CH, –SO_3_, –N–CO–, and CH_2_– groups. These peaks established the formation of PEDOT:PSS on the surface of the Na-Alginate hydrogel. The results indicate that starch from wheat is correlated with Na-Alginate/PEDOT:PSS [26].

### 3.4. Cyclic Voltammetry (CV)

**Figure 6:**
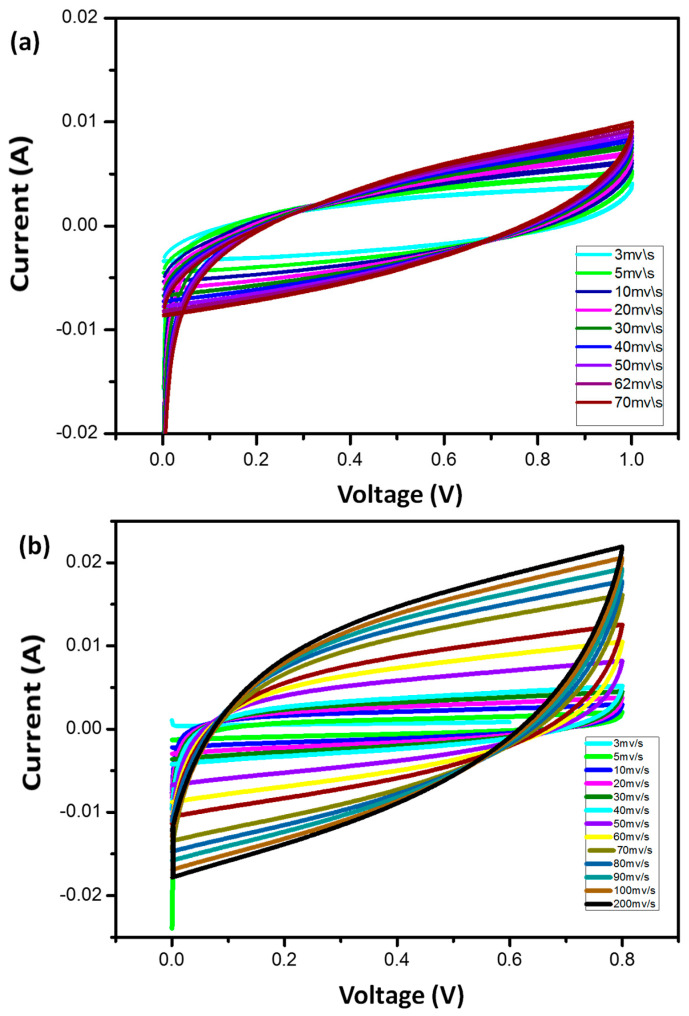
CV curves of (**a**) Na-Alginate hydrogels and (**b**) Na-Alginate/PEDOT:PSS hydrogels at different scan rates of Na-Alginate hydrogels.

In order to assess the stability and capacity of synthesized hydrogel electrolytes, cyclic voltammetry (CV) is a direct current electrochemical method. To determine the potential application of the grown materials for ultra-flexible supercapacitors, the composite hydrogel electrolyte was inserted between the electrodes. The CV measurements of the cells produced using pure hydrogel electrolyte are shown in Figure 6. According to the findings, even at faster scanning rates, all cells kept their rectangular shape. Na-Alginate hydrogel among the cells has a rectangular shape with low scan rates, but with an increase in the scan of more than 70 mv/s, it took on the shape of a leaf. At a higher scan rate, the cell’s maximum specific capacitance, which was measured at 3 mV/s, decreases. Due to the incomplete interaction between the electrodes and the electrolyte as well as the rapid ion movement at the interface between the electrode and the electrolyte, this decrease is noticeable [26].

**Figure 7:**
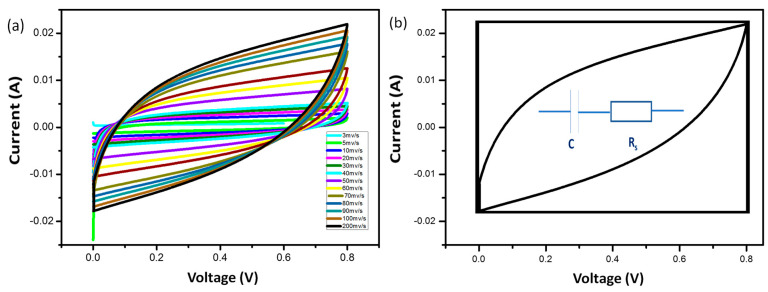
(**a**) CV curves of Na-Alginate/PEDOT:PSS hydrogels as supercapacitor at different scan rates and (**b**) shape of the CV curve at 80 mV/s. Redox peaks are observed only in the case of the use of H_2_SO_4_ acidic.

Figure 7a shows the cyclic voltammetry analysis of the cells fabricated with the composite hydrogel electrolyte. It is worth mentioning that all the cells maintained rectangular shapes even at high scanning rates within the potential window, which indicates that the capacitance source is dominated by the double-layer phenomenon. The specific capacitance of the cell was noted to be 312 F/g at 80 mV/s. The abundance of interstitial porosity in solid hydrogel electrolytes increases the capacity for rapid ionic migration between the aqueous electrolyte and electrode, which allows ions to be rapidly absorbed by cells, hence resulting in excellent quality capacity [27,28]. Additionally, due to the hydrophilic qualities of the composite hydrogel electrolytes, the carbon electrodes are thoroughly wetted and have a high viscosity [28]. The 3D porous structure ensures the diffusion of hydrogel electrolytes into the electrode materials [29].

### 3.5. Galvanostatic Charg–-Discharge (GCD)

**Figure 8:**
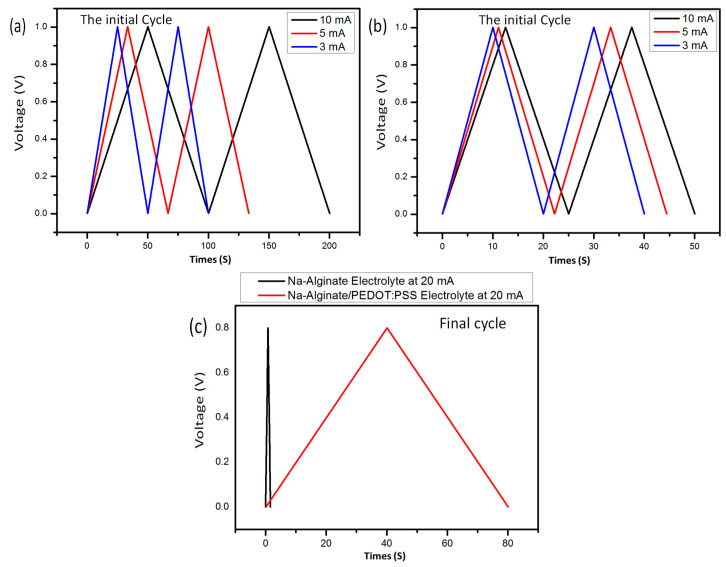
The initial, second, and third cycle of (**a**) Na-Alginate and (**b**) Na-Alginate/PEDOT:PSS hydrogels as supercapacitors at 3, 5 and 8 mA current densities, respectively. (**c**) The final galvanostatic charge–discharge cycle of Na-Alginate and Na-Alginate/PEDOT:PSS Hydrogels at a 20 mA current densities.

Galvanostatic charge–discharge analysis was performed at a current density of 20 mA, and the results are shown in Figure 8a. A supercapacitor with activated carbon as an electrode behaves similarly to the hydrogel. However, when combined with some conductive PEDOT:PSS transition metal oxides, the redox-active materials behave in a way that is ideal for commercial capacitors, storing electric charge through electron transfer or Faradaic reactions [30,31]. In the studied samples, rectangular CV curves or triangular GCDs were observed. It is seen that the capacitor exhibits the redox current peaks on the GCD diagram in a narrow range [32,33]. The GCD curves of all supercapacitors at the current densities between 3 and 10 mA are plotted in Figure 8b,c, which indicates the supercapacitor-type charge storage properties. These results show that Na-Alginate/PEDOT:PSS hydrogels as supercapacitors had a higher capacity retention compared to other devices with Na-Alginate hydrogel electrolytes. This could be due to the large-sized Na-Alginate/PEDOT:PSS electrolyte material with a larger surface area for Faradaic reaction, resulting in the electrode having a higher electrochemical performance [32].

While reversibility (i.e., dissociation) during discharging was not restricted, interactions became sufficiently strong (Figure 8). In this instance, N-doping was accomplished by decreasing Na-Alginate/PEDOT:PSS and treating the resulting nitrogen plasma. However, by taking into account the time spent discharging in both the negative and positive voltage window, the performance was overestimated [33]. However, the capacitance was unmistakably increased compared to the non-N-doped analog. The performance of N-doped activated carbon electrodes was enhanced by combining them with effective crumpling, which prevented the aggregation of reduced Na-Alginate/PEDOT:PSS [32].

Doping with heteroatoms is an alternative method for increasing the capacitance in PEDOT:PSS-based electrolytes and electrodes made of activated carbon electrodes. N-doping in carbons generally improves the wettability and electronic conductivity of the material [30].

As evidenced by redox peaks appearing in the CV in primarily Na-Alginate/PEDOT:PSS hydrogel electrolytes, N-doping has also been reported to induce pseudo-capacitance in carbons. Similar behavior was seen only in H_2_SO_4_ acidic Na-Alginate/PEDOT:PSS hydrogel electrolytes, and it was also seen in N-doped activated carbon. These are thought to only improve the electron conductivity in PEDOT:PSS when there are no free electron lone pairs on the N atoms [31].

Even though redox events were not seen in voltammograms, significant charge storage boosting after N-doping of graphite was reported even in H_2_SO_4_ in the Na-Alginate/PEDOT:PSS hydrogel electrolyte. The authors primarily blamed the prevalence of pyridinic-N configurations for the improved charge storage. When H_2_SO_4_ is dissolved in water, we obtain the hydronium ion H_3_O^+^ and the sulfate ion SO_4_^2−^, and DFT calculations showed that the binding energies of the sulfate SO_4_^2−^ ions with pyridinic vacancies played a crucial role [33,34].

This discovery sparked a hunt for additional functional groups that might improve the capacitance of activated carbon derivatives made using fluorographene chemistry. The porosity of carbon-based materials has a significant impact on their capacitance, as is well known. Consequently, the mounting of substantial and conductive functional groups on activated carbon electrodes has been researched [30,33].

Additionally, because the current is the same for charging and discharging but flows in the opposite direction, the integration of the positive current into the voltage V and the charging and discharging times are the same [35]. The positive current is a linear function of time (E¼Eo + vt) that can give a greater charge than the negative current [32,34]. We also noted that with repeated examination, the GCD diagram remained conservative in the form of a triangle.

**Figure 9:**
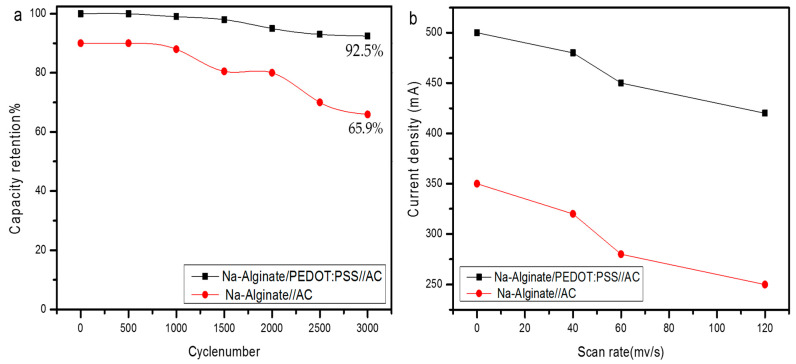
(**a**) Cycling stability of Na-Alginate and Na-Alginate/PEDOT:PSS hydrogel electrolyte/AC supercapacitor after 3000 cycles; (**b**) current density as a function of scan rate.

The cyclic stability of the Na-Alginate and Na-Alginate/PEDOT:PSS hydrogel electrolyte/AC supercapacitor was performed over 3000 charge–discharge cycles at a current density of 10 mA, and the results are shown in Figure 9a. It is seen that the capacity retention of Na-Alginate/PEDOT:PSS/AC slightly increased to 101.6% after 200 charge–discharge cycles, which was due to the activation of the supercapacitor during the beginning cycles. After that, the capacity retention declined gradually to 92.5% after 3000 charge–discharge cycles. In Na-Alginate/AC, the capacity retention declined gradually to 65.9% after 3000 charge–discharge cycles, while tension increased to 89.6%. This shows that the supercapacitor Na-Alginate/PEDOT:PSS/AC had excellent electrochemical stability.

By examining the electrode’s electrochemical kinetics from CV curves, the charge storage mechanism of the Na-Alginate and Na-Alginate/PEDOT:PSS Hydrogels electrolyte/AC supercapacitor was examined. A quasi-linear relationship between anodic and cathodic current densities as a function of scan rate is shown in Figure 9b.

### 3.6. Conductivity of PEDOT:PSS/Alginate

**Figure 10:**
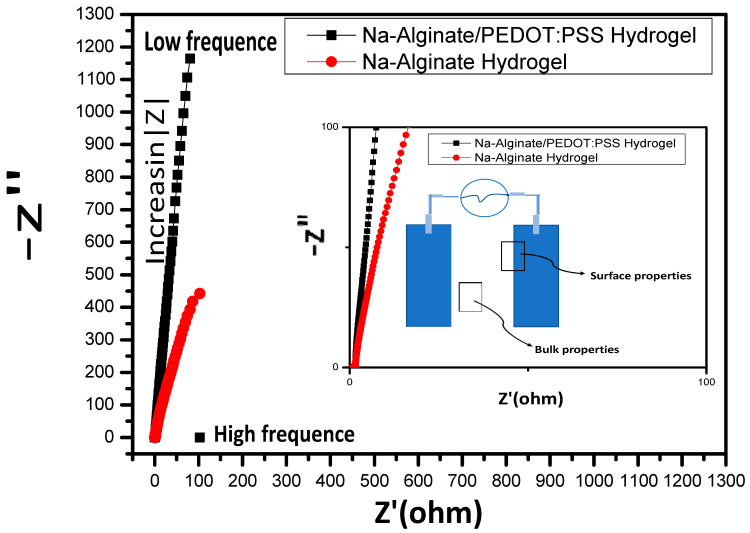
Nyquist plots of the hydrogel electrolytes.

The characteristic electrochemical impedance (EIS) plots of the pure and synthesized hydrogel electrolytes at a temperature of 25 °C are presented in Figure 10. In contrast with the case of solid hydrogel electrolytes consisting of polymers based on ethylene oxide, the Nyquist plot for composite hydrogel shows no semicircle for either sample at high frequency [36], which is due to the low relaxation time (Tg) of the composite, but achieves great mobility at the high-frequency response. For practical battery applications, the electrochemical stability of solid hydrogel electrolytes based on polymers is crucial [37]. The ionic conductivity of Na-Alginate and the composite of Na-Alginate/PEDOT:PSS hydrogels at room temperature was noted as 9.82 × 10^−2^ Scm^−1^ and 7.6 × 10^−2^ Scm^−1^, respectively.

### 3.7. TGA: Thermal Analysis

**Figure 11:**
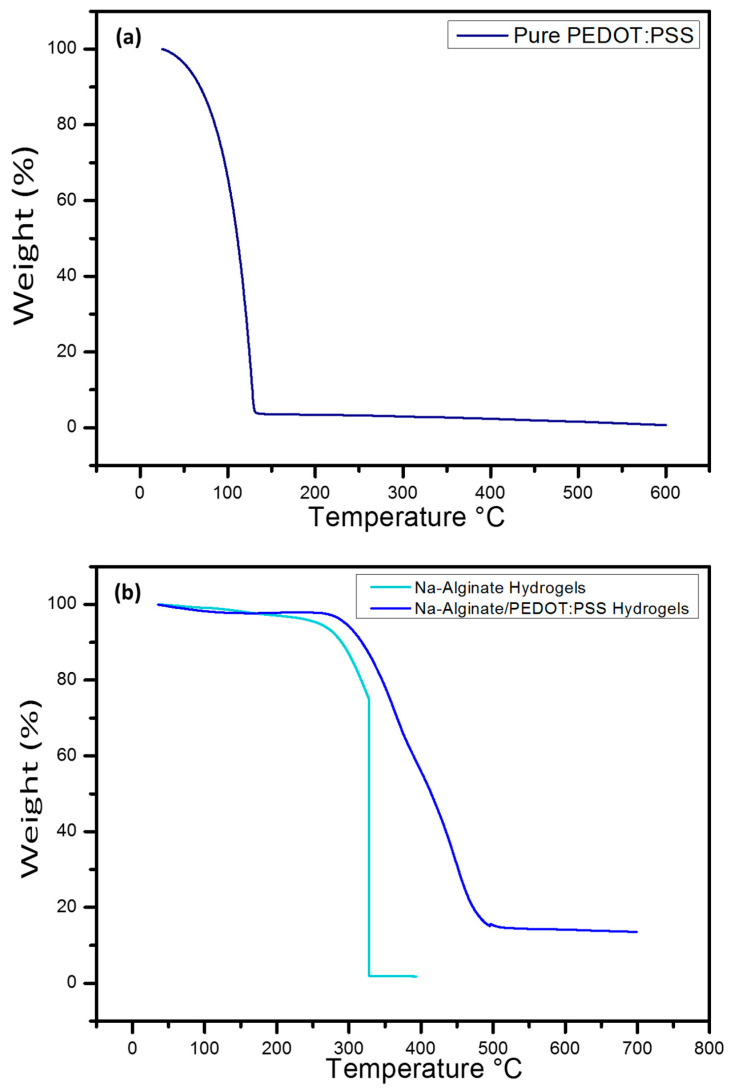
(**a**) TGA of pure PEDOT:PSS and (**b**) Na-Alginate hydrogel electrolyte, Na-Alginate/PEDOT:PSS hydrogel electrolyte.

Figure 11 demonstrates the TGA behavior of pure PEDOT:PSS, Na-Alginate, and Na-Alginate/PEDOT:PSS hydrogels. The results indicate that evaporation of water occurred during the heating process. Pure PEDOT:PSS itself is thermally precarious at temperatures over 100 °C because of the loss of water as shown in Figure 11a. It is seen that the temperature degradation of the Na-Alginate hydrogel occurred at about 230 °C, while for hydrogels with Na-Alginate/PEDOT:PSS, temperature degradation occurred at around 270 °C, as shown in Figure 11b [38,39]. This shows the ability of the grafted adsorbent to withstand high temperatures up to about 335 °C before starting to decompose. Compared to pure hydrogel, the addition of Na-Alginate/PEDOT:PSS to the Na-Alginate hydrogel improved its thermal stability and thus showed less temperature degradation for the reinforced pure hydrogels [40,41]. TGA was divided into three steps. In the first step, evaporation of water occurred between 10 °C and 200 °C, while in the second step, gradual weight loss occurred between 230 °C and 350 °C due to decomposition of hemicellulose, lignin, and cellulose at Na-Alginate, then total degradation occurred at 500 °C [42,43,44].

### 3.8. Self-Healing Property

**Figure 12:**
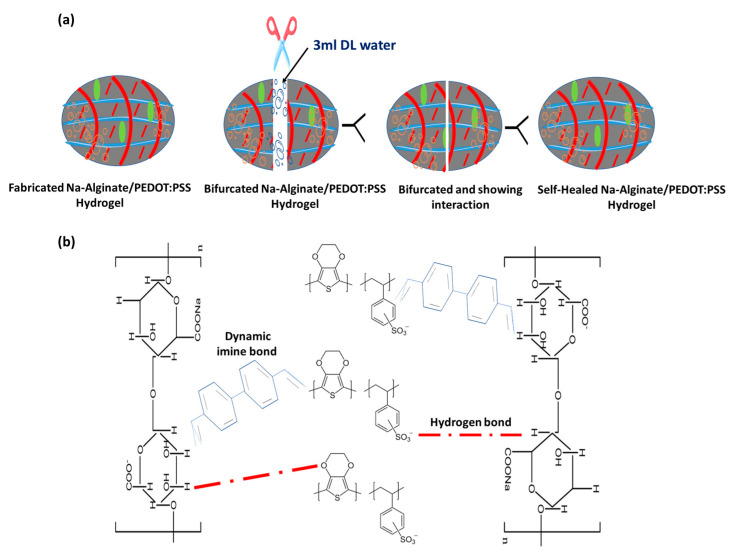
(**a**) Techniques for verifying a hydrogel’s capacity for self-healing. (**b**) A diagrammatic representation of the self-healing mechanism that demonstrates the formation of imine and hydrogen bonds.

Dynamic covalent cross-linkages, covalent interactions, and non-covalent interactions enable self-healing hydrogels to regenerate after sustaining mechanical damage [45]. As a result, interest in the idea of self-healing hydrogels increased. It was inspired by natural organisms, where self-healing properties could be preserved by inducing them through previously extensively discussed interactions or external stimuli [46,47]. These self-healing hydrogels, called “smart hydrogels”, can automatically repair themselves after damage, either fully or partially [48]. The relationship between water and hydrophobic monomers is explained by reversible non-covalent interaction, known as hydrophobic cross-linking [49]. This property was used to design the physically self-healing hydrogels via the self-assembling mechanism of monomers in an aqueous medium. A popular technique for creating hydrophobic bond-based self-healing hydrogels is micellar polymerization [50]. At a temperature of 25 °C, a hydrogel sample was cut into two parts, and then 3 mL DI water was sprayed to wet the surfaces so that the cutting surfaces could stick together easily. They showed a 90% healing efficiency after 50 min of the healing process, and they were gone completely after 90 min. Under physiological conditions, the amine bonds create self-healing hydrogels by creating crosslinks between amine groups and aldehyde or ketone groups of polymers. The Na-Alginate/PEDOT:PSS hydrogel electrolyte’s mechanical strength, adhesion, and self-repair properties are enhanced by the numerous hydrogen bonds that are formed by the two components. The hydrogen bonds on PEDOT:PSS with carboxylate group (-COO) of Na-Alginate, inducing the cross-linking of the hydrogel electrolyte chain, were completely self-healed [49]. Figure 12 shows a diagrammatic of the self-healing property of a hydrogel.

To complete the self-healing process between Na-Alginate and Na-Alginate/PEDOT:PSS, a small amount of 3 mL PEDOT:PSS involved in the Na-Alginate network could effectively enhance the mechanical performance. After 2 h, the Na-Alginate hydrogel exhibited a healing efficiency of 90%. Therefore, such Na-Alginate/PEDOT:PSS hydrogels provide potential platforms for the development of supercapacitor electrolytes.

**Figure 13:**
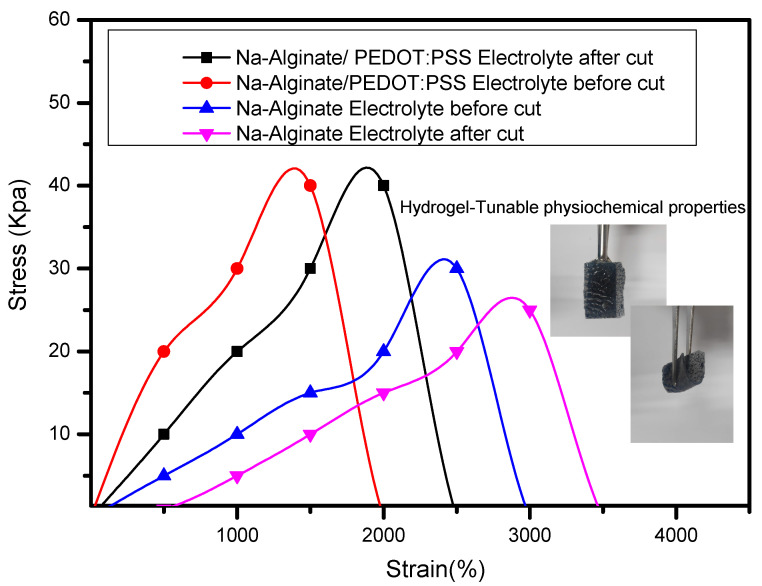
The effects of 3 mL PEDOT:PSS on the self-healing behavior of the hydrogel electrolyte before and after cut hydrogel samples.

The tensile curves for the hydrogels Na-Alginate and Na-Alginate/PEDOT:PSS electrolyte coincided with those of the original samples after re-contacting for two hours, as shown in Figure 13. The tensile curves of pristine hydrogel electrolyte are shown as solid lines, and those of hydrogel after two hours of healing are shown as dashed lines. The Na-Alginate hydrogel’s tensile properties degrade after healing, which is a drawback. The Na-Alginate and Na-Alginate/PEDOT:PSS hydrogels have excellent mechanical properties thanks to the dynamic cross-linking network. After healing, the Na-Alginate hydro-gel that had been chemically cross-linked showed poor tensile properties; its fracture strain and stress were measured at 38.99% and 28.5 kPa, respectively.

In contrast, the Na-Alginate/PEDOT:PSS hydrogel electrolyte was given hydrophobic associations to improve the mechanical properties of the resulting 3 ml PEDOT:PSS, which had a fracture stress of 52 kPa and a fracture strain of 34.51%. This is explained by the PEDOT:PSS’s efficient energy dissipation through destruction and reorganisation. By releasing the divalent ions that cross-link the gel through exchange reactions with PEDOT:PSS, the cross-linked alginate gels can be broken down. Also, Na-Alginate/PEDOT:PSS hydrogels showed excellent elasticity, enabling them to be stretched, twisted, and knotted.

### 3.9. Device Fabrication

**Figure 14:**
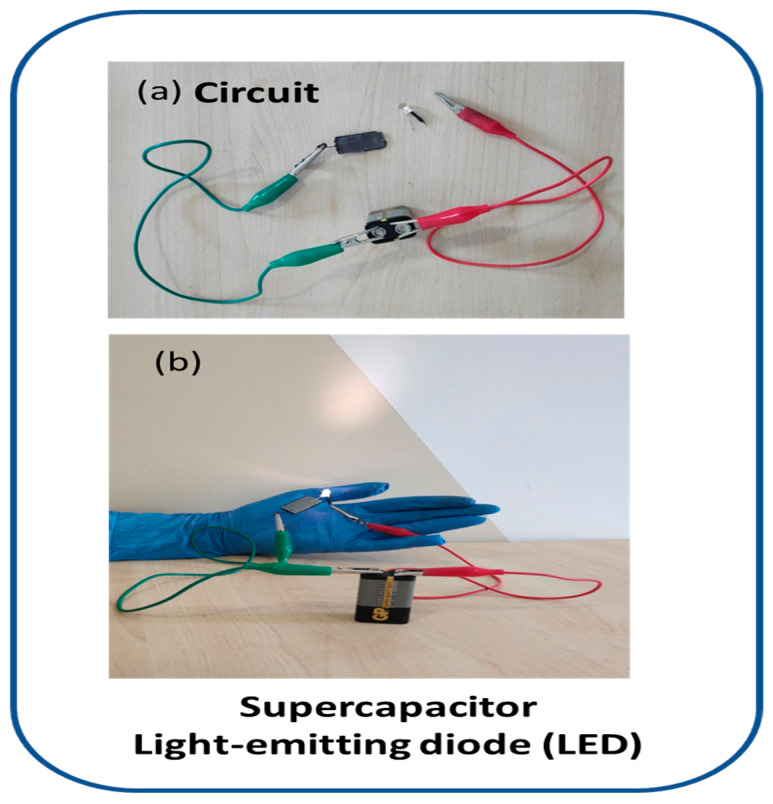
(**a**) Circuit; (**b**) LED is illuminated by Na-Alginate/PEDOT:PSS combined electrolyte hydrogel to fabricate the supercapacitor.

For device fabrication and the testing of the practical application of the device for the supercapacitor, an LED was illuminated by supplying the supercapacitor with a 9-volt battery. The circuit contained a 9-volt battery, a supercapacitor of the composite hydrogel, and an LED lamp connected in series, as shown in Figure 14a. The LED operated with full intensity without any visible damage to the conductive hydrogel as shown in Figure 14b, confirming the stand-alone properties of the hydrogel electrolyte [51,52]. The Na-Alginate/PEDOT:PSS composite electrolyte hydrogel improved the conductivity properties for supercapacitor cell applications [45,52]. The device brilliantly illuminated the LED and transmitted the current through it without causing any damage.

## 4. Conclusions

New composite hydrogel electrolytes based on Na-Alginate/PEDOT were developed as a 3D controllable network for flexible supercapacitor applications. The hydrogel with PEDOT:PSS was successfully formed, according to the FTIR analysis. The PE-DOT:PSS dispersion in the hydrogel was described and revealed in the morphology study as a 3D porous network structure. The electrochemical study revealed that PEDOT:PSS increases ionic conductivity and provides a smooth pathway for the transport of charge carriers and polymers. The ionic conductivity of Na-Alginate and the composite of Na-Alginate/PEDOT:PSS hydrogels at room temperature were noted to be 9.82 × 10^−2^ and 7.6 × 10^−2^ Scm^−1^, respectively. Moreover, Alginate/PEDOT:PSS/AC had excellent electrochemical stability that retained 92.5% of its initial specific capacity after 3000 cycles. The hydrogel responds to electrochemical stimuli in a reversible manner and is electrically stable. The device, which used Na-Alginate/PEDOT:PSS hydrogel as the electrolyte, demonstrated an efficient and favourable response when an LED lamp was lit by providing a strong battery. This excellent performance is due to the hydrogels’ high ionic conductivity, specific capacitance, excellent electrochemical stability, and energy density. The results clearly show that the synthesized hydrogels are promising alternatives for the next generation of supercapacitors and may significantly impact future energy-storage applications.

## Figures and Tables

**Table 1:** Na-Alginate/PEDOT: PSS composite hydrogel electrolytes synthesis scheme.

Formulation	Na-Alginate/PEDOT:PSS	DMSO	H_2_SO_4_
Na-Alginate/PEDOT:PSS composite 1	0.4 g of Na-Alginate + 3 mL PEDOT:PSS	0.2 ML	1 ML
Na-Alginate/PEDOT:PSS composite 2		0.5 ML	2 ML
Na-Alginate/PEDOT:PSS composite 3		1.00 ML	4 ML

## Data Availability

The datasets generated during and/or analyzed during the current study are available from the corresponding author upon reasonable request.
